# Women’s holistic self-care behaviors during pregnancy and associations with psychological well-being: implications for maternal care facilities

**DOI:** 10.1186/s12884-022-04961-z

**Published:** 2022-08-09

**Authors:** Lam Duc Nguyen, Long Hoang Nguyen, Ly Thi Ninh, Ha Thu Thi Nguyen, Anh Duy Nguyen, Linh Gia Vu, Hao Si Anh Nguyen, Son Hoang Nguyen, Linh Phuong Doan, Thuc Minh Thi Vu, Bach Xuan Tran, Carl A. Latkin, Cyrus S. H. Ho, Roger C. M. Ho

**Affiliations:** 1 Department of Anaesthesiology, Hanoi Medical University, Hanoi, Vietnam; 2 Department of Global Public Health, Karolinska Institutet, Stockholm, Sweden; 3 Social Affair Department, Ca Mau Obstetrics & Pediatrics Hospital, Ca Mau, Vietnam; 4 Hanoi Obstetrics and Gynecology Hospital, Hanoi, Vietnam; 5 Institute for Global Health Innovations, Duy Tan University, Da Nang, Vietnam; 6 Faculty of Nursing, Duy Tan University, Da Nang, 550000 Vietnam; 7 Institute of Health Economics and Technology, Hanoi, Vietnam; 8 Center of Excellence in Evidence-Based Medicine, Nguyen Tat Thanh University, Ho Chi Minh City, Vietnam; 9 Institute for Preventive Medicine and Public Health, Hanoi Medical University, Hanoi, Vietnam; 10 Bloomberg School of Public Health, Johns Hopkins University, Baltimore, MD USA; 11 Department of Psychological Medicine, Yong Loo Lin School of Medicine, National University of Singapore, Singapore, Singapore; 12 Institute for Health Innovation and Technology (iHealthtech), National University of Singapore, Singapore, Singapore

**Keywords:** Maternal behavior, Psychological well-being, Social support, Pregnant women, Structural equation modeling

## Abstract

**Background:**

Self-care behaviors during pregnancy significantly impacts mother and children's health. This study aimed to explore the self-care behaviors and the associations of these behaviors with the psychological well-being of women during pregnancy, as well as the mediating effects of different social support with these associations.

**Methods:**

A cross-sectional data of 562 pregnant women at Hanoi Obstetrics & Gynecology Hospital and Ca Mau Obstetrics & Pediatrics in Vietnam were analyzed. Questions about self-care behaviors, pregnancy characteristics, social support, and psychological well-being were asked. Multivariate regression models were performed. Structural Equation Modeling (SEM) was employed to test the mediating effects of different social support with the association between self-care behaviors and psychological well-being.

**Findings:**

Only 13% of pregnant women often or always did physical exercise at least three times a week, and 40% consumed enough fiber and five servings of vegetables a day. Only 78.7% always avoided alcohol drinking, and 53.9% of pregnant women avoided being exposed to second-hand smoking and 71,7% avoided using traditional medicine without physicians’ prescriptions. Around 66% of pregnant women always or often had prenatal care checkups as scheduled. Information sources, social support and childbirth expectation were major drivers for self-care practices. SEM model showed that social support mediated the relationship between maternal health behaviors and mental well-being.

**Conclusion:**

This study highlighted the remarkable gaps in self-care practices among our pregnant women, which were significantly associated with their mental well-being. Social support-oriented consultancy and interventions should be warranted for improving behaviors and the mental well-being of pregnant women in Vietnam.

## Introduction

Prenatal behaviors are major attributable to the success of pregnancy. World Health Organization (WHO) recommends that pregnant women should be consulted about health behaviors such as healthy diet, physical activity, daily intake of food supplements, and avoidance of substance use and abuse before pregnancy [1]. Healthy practices positively affect the health condition of pregnant women and the development of their offspring [2], while unhealthy behaviors can result in many physical and psychological consequences, as well as increasing the risk of birth defects, miscarriage or preterm birth [3–6]. However, unhealthy practices are prevalent while healthy behaviors are not sufficient in this population. For example, prior meta-analyses estimated that approximately 10% of pregnant women used alcohol [7], 0.8 to 8.1% smoked tobacco [8], and 60% consumed low-energy diets [9]. Low education, low socioeconomic status, unemployed, poor social support or unplanned pregnancy were associated with a higher likelihood of being engaged with unhealthy practices [10–13]. Meanwhile, only 15% of pregnant women adhered to recommendations for physical activity [14] and 3% followed recommended diets with four food groups [15].

A holistic approach to health considers multidimensional factors of well-being, including physical, mental, emotional, social, intellectual as well as spiritual. Therefore, besides practicing healthy physical behaviors, having good psychological wellness is an important part of the pregnancy period since it is associated with pregnancy outcomes. Psychological stress and prenatal depression in pregnant women in both short and long terms cause an imbalance in homeostasis and weaken the body’s immune responses, raising the risk of preeclampsia, preterm birth or miscarriage [16, 17]. In addition, poor psychological well-being during the prenatal period is a significant predictor of postpartum depression [18] as well as associated with various adverse outcomes in children [19]. In literature, health behaviors have bidirectional relationships with the psychological well-being of pregnant women [20]. Women with unhealthy lifestyles tend to simultaneously experience mental health problems such as depression and/or anxiety [21]; whereas, women exhibiting more depressive symptoms were more likely to engage in unhealthy behaviors (e.g. tobacco smoking, alcohol drinking or cocaine using) [22]. Further investigation into such a relationship would potentially provide insights for improving the mental wellness of pregnant women and consequently the health condition of the mothers and their children.

Social support has a critical role in changing lifestyles and improving both physical and psychological health outcomes in mothers [23]. Previous studies revealed the influences of social support on the success of pregnancy and mothers’ postnatal health conditions through assisting them in maintaining psychological wellbeing [24, 25], feeling less anxious [26–29], or reducing stress [30, 31]. Lower social support of pregnant women was associated with lower birth weight infants [32], worse labor progress, and babies with lower Apgar scores than women with higher social support [33]. Existing literature also reported the role of social support mediating the relationship between depression and birth outcomes. Depressed women receiving lower social support gave birth to babies with lower Apgar scores than those with higher social support [34]. Social support has been shown to have a similar buffering impact on birth weight among women experiencing stress [35]. In addition, pregnant women having high satisfaction with their marital relationship are more likely to have healthy diets [36]. However, whether social support buffers associations between health behaviors and psychological well-being of pregnant women have not been sufficiently explored. Understanding this mediating effect is critical for designing interventions to improve pregnancy care. This study aimed to examine health behaviors among pregnant women and their associated factors, as well as the mediating effects of social support on the relationships between health behaviors and psychological well-being in this population.

## Methods

### Study design and participants

A cross-sectional study was conducted in two obstetric medical centers including Hanoi Obstetrics & Gynecology Hospital and Ca Mau Obstetrics & Pediatrics Provincial Hospital from January to February 2021. Criteria for selecting participants as follows: (1) Aged 18 and older; (2) Agree to participate in research; (3) Being pregnant and not being delivered at the time of the study; (4) Did not have any cognitive impairment or any disabilities which might limit the ability to answer the interview questionnaire.

We computed the sample size by using the formula for estimating a population proportion with relative precision, including the following parameters: confidence level α = 0.05, expected proportion = 0.15 (the proportion of pregnant women adhered to physical activity recommendations [14]), and relative precision = 0.2. The necessary sample size was 545 pregnant women. We added 10% of the sample size for preventing drop-out, resulting in 600 pregnant women for the final sample size. The sampling process was performed parallel in both hospitals. The pregnant women were conveniently approached and recruited to this study when they visited the antenatal clinics for regular care. At the end of the study period, a total of 675 women were recruited. After excluding those not completing questions about health behaviors, psychological well-being and social support, the final sample size for analysis was 562 (completion rate 83.3%). The Institutional Review Board of Hanoi Obstetrics & Gynecology Hospital granted the study protocol (Code: 07 QĐ/PS‐TTĐT CĐT).

### Data collection and measurement

We developed a structured questionnaire and used face-to-face interviews for collecting data. Participants were initially screened from the eligibility criteria before being invited to be enrolled in the study. Then, women agreeing to participate would be invited to a private room for the interview. We firstly informed a short introduction about the study and participants’ rights and benefits. Next, the data collector (i.e. trained nurses or undergraduate medical students) interviewed them by using the questionnaire. Each interview lasted 15–20 min. The structure of the questionnaire included: 1) Demographic information; 2) Pregnancy characteristics; 3) Health behaviors during pregnancy; 4) Psychological wellbeing, and 5) Social support.

#### Health behaviors

In this study, we asked pregnant women to report the frequency of fifteen recommended health behaviors for pregnancy that they performed during the pregnancy period. Each behavior had five options for response: 1 = None; 2 = Rarely; 3 = Sometimes; 4 = Often; and 5 = Always.

#### Psychological well-being

The World Health Organization-5 Well-being scale was utilized to assess participants’ mental well-being in the last two weeks [37]. Participants were asked to respond to five questions about different psychological aspects, using a 6-point Likert scale ranging from 0 (at no time) to 5 (all of the time). The total score was calculated and converted to a new transformed score from 0 to 100, which a higher score meant a higher level of psychological well-being. The results of previous studies have discovered that the Cronbach’s alpha of The World Health Organization-5 Well-being scale was 0.85 [38].

#### Social support

The Perinatal Infant Care Social Support (PICSS) instrument was used to evaluate social support [39]. Participants were asked to rate how strongly they agreed with twenty-two statements using a 4-point Likert scale from 1 “Totally disagree” to 4 “Totally agree”. This scale measured social support in four subscales: Informational support (7-item scores from 7 to 28); Instrumental support (7-item scores from 7 to 28); Emotional support (4-item scores from 4 to 16); Appraisal support (4-item scores from 4 to 16). The Cronbach’s alpha was excellent at 0.9727.

#### Demographic and pregnancy characteristics

Demographic characteristics such as age, education, occupation, health insurance status, living arrangement, partner’s age, partner’s education, and monthly household income. Pregnancy characteristics consisted of complications of pregnancy, source of maternal care information (health professional, internet/social network, friends/relatives, radio & television, smartphone application, newspapers & book, phone message, or poster/banner). Expectancy of having a baby and fear of childbirth were asked with 10-point rating scales from 0 “No having expectation” or “No fear” to 10 “Very strong expectation” or “Extreme fear”, respectively.

### Data analysis and statistical method

Data management and analysis were performed using Stata version 15.0 software (Stata Corporation). A listwise deletion approach was used to handle the missing data. A significance level of *p* < 0.05 was used. The Chi-square test for categorical variables and the Mann–Whitney test for continuous variables were used to make comparisons between urban and rural pregnant women. To investigate the scale of items and enhance the interpretability of this research, the exploratory factor analysis was employed to define factors with a threshold of an eigenvalue of 1.6 by scree test, in which the curve had been flattened. An orthogonal varimax rotation with Kaiser normalization was used. A value of 0.29 was chosen as the cut-off point for factor loading. The result of the Kaiser–Meyer–Olkin test was 0.7676, suggesting that the sample size was adequate for EFA. The p-value of Bartlett’s Test of Sphericity was less than 0.01 (χ2 = 3873.431; Degrees of freedom = 105; *p*-value = 0.000), which indicated that EFA was helpful for restructuring the health behavior scale. Cronbach's alpha was used to assess the internal consistency of each factor. The score of each factor was calculated by summing the score of all items within the factor and then dividing it by the number of items in this factor. The score ranged from 1 to 5, which a higher score indicated a higher level of behavior engagement.

Multivariate Tobit regression models were carried out to explore the association of health practices demographic characteristics, pregnancy characteristics, and social support. To minimize the models, stepwise forward selection strategies were used with a log-likelihood ratio test at a p-value of 0.2. Structural equation modeling (SEM) was utilized to examine the mediating effect of social support between health behaviors and psychological well-being, adjusting for age and pregnancy status. For obtaining both direct and indirect/mediated directions, the SEM builder employs the maximum likelihood method. The built SEM model then was evaluated by goodness-of-fit indices involving Root Mean Square Error of Approximation (RMSEA), Comparative Fit Index (CFI), Standardized Root Mean Square Residual (SRMR).

## Results

Demographic characteristics are shown in Table 1. A total of 562 pregnant women participated in the study. The mean age of women was 29.1 years old (SD = 5.3). More than half of the sample lived in an urban setting, and 85.3% of pregnant women in urban settings came from the North of Vietnam. The mean monthly income of households in urban was above two times higher than that of households in rural. Two-third of pregnant women in urban areas lived with partners, yet this rate in rural areas was only 48.6%.

**Table 1:** Demographic characteristics of respondents

Characteristics	Living location					
	**Urban**		**Rural**		**Total**	
	**N**	**%**	**N**	**%**	**N**	**%**
**Total**	313	55.7	249	44.3	562	100.0
**Education level**						
Secondary or below	12	3.8	62	25.0	74	13.2
High school	54	17.3	136	54.8	190	33.9
College/University	69	22.1	16	6.5	85	15.2
Post graduated	177	56.7	34	13.7	211	37.7
**Occupation**						
Farmer, worker	16	5.1	64	25.7	80	14.2
Public servant	34	10.9	15	6.0	49	8.7
Office worker	154	49.2	14	5.6	168	29.9
Housewife	53	16.9	127	51.0	180	32.0
Others	56	17.9	29	11.6	85	15.1
**Type of insurance**						
Private health insurance	128	28.1	309	78.6	437	51.5
Voluntary health insurance	334	73.2	85	21.6	419	49.4
**Partner’s education level**						
Secondary or below	13	4.2	28	11.3	41	7.3
High school	43	13.7	112	45.2	155	27.6
College/University	82	26.2	84	33.9	166	29.6
Post graduated	175	55.9	24	9.7	199	35.5
**Living arrangements**						
Parents in law	117	37.4	118	47.4	235	41.8
Parents	28	8.9	25	10.0	53	9.4
Partner	213	68.1	121	48.6	334	59.4
**Region**						
North	267	85.3	54	22.0	321	57.4
South	46	14.7	192	78.0	238	42.6
	**Mean**	**SD**	**Mean**	**SD**	**Mean**	**SD**
Age (years)	29.4	4.5	28.7	6.1	29.1	5.3
Partner’s age (years)	32.5	5.0	30.8	6.3	32.0	5.6
Monthly household income (USD)	762.3	621.9	343.8	197.3	614.8	551.3

Table 2 shows that pregnant women accessed various sources of information and there were significant differences in these sources between women in the urban areas and rural areas. To women in urban settings, the internet/social network was the main source of information (76.4%), while women in rural areas received information mostly from health providers (83.5%). Phone messages and posters/banners were not popular choices with only 7.8% and 5.3% of pregnant women receiving information from those sources, respectively. The expectation of having a baby in this study was high with a mean score of 7.8 (SD = 2.1, range score 0–10), and there was a significant difference between pregnant women of different areas (*p* < 0.01). The mean score of fear of childbirth among pregnant women in urban areas was 5.7 (SD = 2.9); which was significantly higher than those in rural settings (mean = 5.3, SD = 1.5) (p < 0.01). The mean score PICSS Informational support, PICSS Instrumental support, PICSS Emotional support, PICSS Appraisal support was 21.6 (SD = 2.7, range score 7–28), 21.5 (SD = 2.7, range score 7–28), 12.3 (SD = 1.5, range score 4–16), and 12.3 (SD = 1.4, range score 4–16), respectively.

**Table 2:** Characteristics of pregnancy experience

Characteristics	Living location						*p*-value
	**Urban**		**Rural**		**Total**		
	**N**	**%**	**N**	**%**	**N**	**%**	
**Ever having complications of pregnancy**	88	28.1	29	11.6	117	20.8	< 0.01
**Source of information about maternity care**							
Health professional	154	49.2	208	83.5	362	64.4	< 0.001
Internet/Social network	239	76.4	119	47.8	358	63.7	< 0.001
Friends/Relatives	188	60.1	124	49.8	312	55.5	0.015
Radio, television	57	18.2	93	37.3	150	26.7	< 0.001
Smartphone application	92	29.4	53	21.3	145	25.8	0.01
Newspapers, book	74	23.6	39	15.7	113	20.1	0.019
Phone message	25	8.0	19	7.6	44	7.8	0.876
Poster/Banner	12	3.8	18	7.2	30	5.3	0.075
	**Mean**	**SD**	**Mean**	**SD**	**Mean**	**SD**	
Expectation of having a baby (0–10)	8.9	1.8	6.5	1.8	7.8	2.1	< 0.001
The fear of childbirth (0–10)	5.7	2.9	5.3	1.5	5.5	2.4	0.004
PICSS^*^ Informational support (7–28)	21.8	3.2	21.5	2.0	21.6	2.7	0.081
PICSS^*^ Instrumental support (7–28)	21.6	3.1	21.4	2.0	21.5	2.7	0.951
PICSS^*^ Emotional support (4–16)	12.4	1.7	12.2	1.2	12.3	1.5	0.402
PICSS^*^ Appraisal support (4–16)	12.3	1.6	12.2	1.1	12.2	1.4	0.573

^*^ *PICSS* Perinatal Infant Care Social Support

The construct validity and reliability of maternity care practice from pregnant women are reported in Table 3. From factor analysis, three dimensions were reclassified namely “Healthy behaviors”, “Risk behavior avoidance” and “Health-seeking behaviors”. Cronbach’s alpha was accepted across domains, ranging from 0.60 to 0.80.

**Table 3:** Factor loadings of maternity care practice

Items	Healthy behaviors	Risk behavior avoidance	Health Seeking behaviors
1. Avoid alcohol use		0.58	
2. Avoid tobacco use		0.87	
3. Avoid secondhand smoke		0.39	
4. Avoid addictive substances such as drugs		0.84	
5. Avoid using traditional medicine not according to the instructions of a doctor		0.33	
6. Physical activity, exercise at least 3 times/week	0.29		
7. Take vitamins and minerals as recommended	0.60		
8. Take iron and folic acid as recommended	0.78		
9. Take calcium as recommended	0.71		
10. Eat at least 5 servings of vegetables/day	0.65		
11. Eat enough fiber daily	0.76		
12. Consult medical staff about maternity care			0.76
13. Ask health-care workers about incomprehensible information in maternity care			0.77
14. Discuss with health professionals about the effects of medication on pregnancy and fetus			0.68
15. Maternity check-up according to schedule			0.74
**% floor**	0.7	44.1	0.9
**% ceiling**	0.2	0.0	0.9
**Reliability**			
Cronbach’s alpha	0.80	0.60	0.73
**Domains scores**			
Mean	3.5	4.7	3.2
SD	0.6	0.4	0.6

**Fig. 1:**
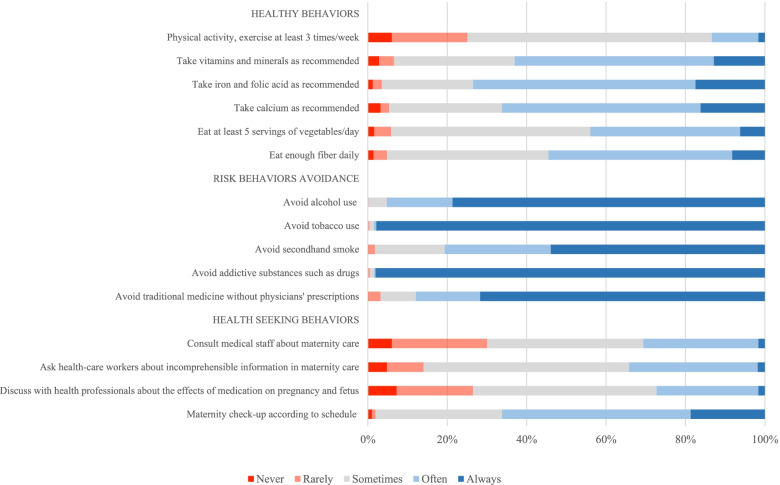
Frequency of different health behaviors

Figure 1 shows different health behaviors of pregnancy. More than two-thirds of pregnant women often or always took vitamins and minerals, iron and folic acid, and calcium as recommended. However, only 13% of pregnant women often or always did physical exercise at least three times a week. The rates of pregnant women taking enough fiber a day and five servings of vegetables a day were more than 40%. More than 90% of pregnant women always avoided substance uses such as tobacco smoking and addictive substances, but only 78.7% always avoided alcohol drinking. There was only about 53.9% of pregnant women avoided exposing second-hand smoking and 71,7% avoided using traditional medicine without physicians’ prescriptions. Around 66% of pregnant women always or often had prenatal care checkups as scheduled. There were only approximately 30% of women who often or always consulted medical staff for their maternal health and 27% of women who often or always discussed with health staff about maternity care.

Table 4 reveals that a higher level of healthy behaviors practices was positively associated with a higher expectation of having a child (Coef. = 0.05, 95%CI = 0.00–0.10) and higher appraisal support (Coef. = 0.05; 95%CI = 0.00–0.09), but negatively related to the fear of childbirth (Coef. = -0.03; 95%CI = -0.03-—0.01). In terms of risk behaviors avoidance, high education, living with partners, and considering relatives/radio, television/internet, social networks were associated with lower levels of risk behaviors avoidance. Meanwhile, a higher level of risk behaviors avoidance practices was positively associated with the Southern region (Coef. = 0.38, 95%CI = 0.08–0.69), high expectation of having a child (Coef. = 0.08; 95%C = 0.03–0.14), and high appraisal support (Coef. = 0.06, 95%CI = 0.01–0.11).

**Table 4:** Factors associated with health behaviors among pregnant women

Variables	Healthy behaviors	Risk behaviors avoidance	Health seeking behaviors
	**Coef. (95% CI)**	**Coef. (95% CI)**	**Coef. (95% CI)**
**Demographic**			
**Education** (ref: Secondary or below)			
High school	-0.12 (-0.27; 0.04)	-0.28** (-0.52; -0.03)	
College	-0.05 (-0.25; 0.14)	-0.28** (-0.55; -0.00)	
Post graduated	0.10 (-0.11; 0.30)	-0.28* (-0.56; 0.01)	
**Having private health insurance (Yes vs No)**	-0.10 (-0.23; 0.04)	0.15* (-0.01; 0.32)	
**Living with parents in law (Yes vs No)**	0.07 (-0.02; 0.16)		
**Living with partner (Yes vs No)**		-0.18*** (-0.30; -0.05)	-0.24*** (-0.37; -0.11)
**Husband’s age (Unit: year)**			0.01** (0.00; 0.03)
**Husband’s education** (ref: Secondary or below)			
High school	-0.01 (-0.21; 0.19)	-0.11 (-0.37; 0.14)	-0.01 (-0.26; 0.24)
College	0.12 (-0.08; 0.31)	0.32** (0.06; 0.57)	0.08 (-0.15; 0.32)
Post graduated	0.15 (-0.06; 0.36)	0.18 (-0.09; 0.44)	0.23* (-0.01; 0.46)
**Region** (ref: North)			
South	-0.22* (-0.46; 0.01)	0.38** (0.08; 0.69)	-0.56*** (-0.73; 0.38)
**History of maternity care**			
**Maternity problem (Yes vs No)**		-0.08 (-0.21; 0.05)	0.14** (0.02; 0.26)
**Source of information about pregnancy care (Yes vs No)**			
Health professional		0.10 (-0.04; 0.24)	0.21*** (0.06; 0.36)
Internet/Social network		-0.15** (-0.30; -0.00)	
Relatives		-0.23*** (-0.36; -0.10)	-0.09 (-0.22; 0.04)
Radio, television	-0.06 (-0.18; 0.07)	-0.29*** (-0.46; -0.13)	
Smartphone application		-0.08 (-0.21; 0.05)	
Poster/Banner		-0.36* (-0.75; 0.03)	
**Prenatal preparation**			
The expectation of having a child (Unit: score)	0.05** (0.00; 0.10)	0.08*** (0.03; 0.14)	
The fear of childbirth (Unit: score)	-0.03*** (-0.04; -0.01)		
**Social support**			
Informational support (range: 7–28) (Unit: score)	0.02 (-0.00; 0.04)	-0.02 (-0.04; 0.00)	
Instrumental support (range: 7–28) (Unit: score)			0.02** (0.00; 0.04)
Appraisal support (range: 4–16) (Unit: score)	0.05** (0.00; 0.09)	0.06** (0.01; 0.11)	

^***^ p < 0.01, ** p < 0.05, * p < *0.1*

*° A higher score means less risky behavior was performed*

Regarding health-seeking behaviors, living with partners and in the Southern region was negatively related to these practices; whereas, higher husband’s age, having maternity problems, considering health professionals as information sources and high instrumental support were positively associated with health-seeking behavior practices.

**Fig. 2:**
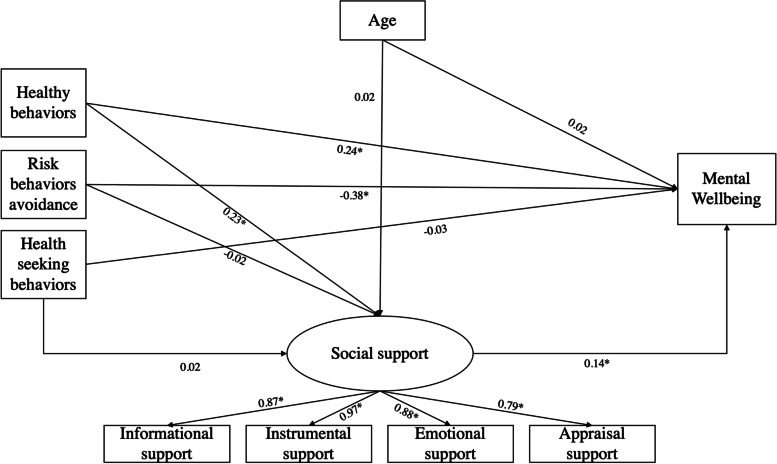
Structural model and standardized path coefficients (*n* = 495). Note: (* *p* < 0.05)

Figure 2 illustrates the SEM model to show the mediating effects of social support on the associations between self-care practices and the mental well-being of pregnant women. The goodness-of-fit indices were acceptable with RMSEA = 0.153, CFI: 0.903, SRMR = 0.030. The model showed healthy behaviors and risk behavior avoidances were significantly associated with the mental well-being of pregnant women. Social support was found to have a positive relationship with mental well-being.

The estimations of the models, direct and indirect SEM paths and 95% Confident interval are presented in Table 5. Social support was only found to improve the relationship between healthy behaviors and mental well-being. The indirect effect of social support accounted for 11.9% of the total effect and 13.5% of the direct effect.

**Table 5:** Indirect effects for maternity care practice and mental wellbeing mediated by social support

Pathways	Indirect effect	95% Confident interval	Total effect/ % Total effect	Direct effect/ % Direct effect
Healthy behavior/Social support/ Mental wellbeing	0.032*	0.009;0.055	0.268/11.9%	0.236/13.5%
Risk behavior/Social support/ Mental wellbeing	-0.003	-0.015; 0.010	0.381/0.7%	0.378/0.7%
Maternity health services/Social support/Mental wellbeing	0.003	-0.012; 0.017	0.026/9.9%	0.029/9.0%

^*^p < *0.05*

## Discussion

This study suggested substantial gaps in health behaviors among pregnant women in Vietnam. While the frequency of risk behaviors such as alcohol use or second-hand smoking exposure was relatively high, the performance of healthy behaviors such as physical activity, vegetables/vitamins, and mineral supplements consumption was insufficient. This study also revealed the potential role of social support in improving their psychological well-being via mediating effects of health behaviors.

In the current study, we found that the health behaviors among Vietnamese pregnant women were critical health issues. The rates of risky behaviors such as alcohol use, tobacco use and second-hand smoking exposure in our sample were relatively similar or higher when compared to the previous findings in Vietnamese women in general and pregnant women in particular [40, 41]. Moreover, the result indicated approximately a third of our pregnant women used traditional medicine or herbal medicine without physicians’ prescriptions. It should be noted that the efficiency and effectiveness of traditional medicine, especially among pregnant women, were still limited [42]. Moreover, the usage of traditional medicine in Vietnam was frequently used without scientific studies [43], and there was a common belief that herbal medicine possessed no adverse effects [44]. Therefore, there is a need to increase the knowledge of pregnant women of self-medication to prevent side effects of traditional medicine on mothers and unborn children. In addition, the prevalence of healthy behavior among pregnant women was lower than in previous studies. The rate of physical activity during pregnancy (women who often or always did exercise at least 3 times a week) was around 12%. The result of this study was in line with that in Brazil (13.4% in the third trimester) [45], but lower than the level of physical activity of Chinese pregnant women (57.1%) [46]. The reason might be most pregnant women in this study was housewife and office worker, they tended to spend more time on housework rather than doing exercise or sport [47]. Moreover, it should be noticed that the questionnaire used in this study mentioned only physical exercise but not domestic work or work activities. Additionally, the rates of taking iron and folic acid were higher compared to a previous study conducted in Vietnam (25.8%) [48]. This result was similar to that reported in China (66.7%) [49], but higher than that of Japan (20.5%) [50], and Korea (10.5%) [51].

More than 60% of pregnant women visited maternity as scheduled. However, a similar prevalence of women did not consult or ask health care workers about maternity care. It could be justified that our samples used the internet/social network as one of the most common sources of information. Due to the rise of internet access over the last decade, many women have access to a wide variety of pregnancy, birth and parenting information [52]. The privacy, accessibility, and breadth of information available on the internet might be reasons why women prefer the Internet to other sources of information [53–55]. Women could use the internet to improve their comprehension of knowledge offered by their maternity care provider or to decide whether they should pursue additional advice [54]. Notably, the use of internet/social networks, relatives and radio/television as the information sources decreased the level of risk behavior avoidance practice. These informal sources were raised concerns about the accuracy of available information [52]. Women may have a lack of skills to appraisal information they found from these sources [52]. Despite health professionals as the main source of information for major pregnant women, we found only an association between this information source and health-seeking behaviors, why no relationship was found between this source and the other two behavior groups. This issue could be justified by two explanations: 1) There was homogeneity in other behaviors between the groups with and without using health professionals as a source of information; hence, the study could only find differences in health-seeking behaviors; or 2) Pregnant women in this study did not practice healthy behaviors or risks behavior avoidance according to the health professionals’ recommendations, raising a question about the role of health workers in providing maternal health information. Longitudinal follow-up studies should be conducted in the future to assess the role of health professionals in the self-care practices of pregnant women in Vietnam.

In this study, childbirth expectation among pregnant women was associated with the increase of the level of healthy behavior practices, and risk behavior avoidance, which might be explained by the maternal–fetal relationship [56]. We also found that fear of childbirth decreased the level of healthy behavior practices. It should be explained by the physiological side-effect of pregnancy (e.g., muscle pain, or backaches, which might hinder them to practice healthy behaviors such as physical activities. Besides, our result was consistent with the previous study, which confirmed that social support affected health-related behaviors such as smoking or dietary habits [57] and was a protective factor for pregnant women [57, 58]. In this study, appraisal support was related to both healthy behavior and risk behavior avoidance practices. Pregnant women were encouraged to turn one another for appraisal support in the form of sharing experiences, appreciating her childcare skills, understanding her need for help, and receiving feedback from health providers about her childcare skills. This result was in line with the previous study, which confirmed appraisal support had a positive impact in reducing risky behaviors among pregnancy [59, 60].

In the current research, healthy behaviors improved the mental well-being of pregnant women, which was consistent with a previous study [61]. With mediating effect of social support, the relationship between these variables was stronger. However, we also found out that risky behavior avoidance decreased mental well-being. It should be noted that, in our sample, more than 50% of pregnant women avoided being exposed to second-hand smoking. The source of smoking mainly came from restaurants, cafeterias, homes or workplaces [62]. We supposed that finding a smoke-free restaurant, asking husband or father-in-law or coworkers not to smoke caused pressure for pregnant women. However, social support helped mediate this relationship and increase mental well-being. Social support may indirectly increase maternal mental well-being by acting as a buffer against potential adverse effects of stressful events [23].

Several important implications could be drawn from study findings. First, our results suggest that there is a need to improve health literacy between healthcare providers and pregnant women. Communication is not just a one-way but an active social process. Besides, our findings point to a need to introduce reliable information sources (e.g. websites, television channels, etc.) with guidance from health experts to ensure that pregnant women received accurate information. Second, interventions to mitigate the influences of negative behaviors on the mental well-being of pregnant women should integrate social support as the main component to cope with stressful situations. Meanwhile, in non-stressful situations, social support would provide a positive personal emotional experience.

The strength of our study lies in the large sample size of 562 pregnant women, which increased the generalizability of the findings. Moreover, as we developed survey questions based on standardized scales, the reliability of analyses was substantially improved. We acknowledged several possible limitations of our review. First, as our study design was cross-sectional, it is unable to conclude causality between risk factors and outcomes. Second, recall bias and social desirability response bias might be caused by the self-reported questionnaire. Third, the convenience sampling procedure used to select pregnant women was prone to bias. Moreover, we only obtained data from pregnant women seeking antenatal care at two hospitals; thus, our survey might not be completely representative of all pregnant women in Vietnam. Finally, while gathering quantitative statistics on a variety of health behaviors, we did not compile evidence on all aspects of pregnant women's lifestyles, for example, protein consumption or nutrition education.

## Conclusion

To conclude, this study highlighted the remarkable gaps in self-care practices among our pregnant women, which were significantly associated with their mental well-being. Information sources, social support and childbirth expectation were major drivers for self-care practices. Social support-oriented consultancy and interventions should be warranted for improving behaviors and the mental well-being of pregnant women in Vietnam.

## Data Availability

The data that support the findings of this study are available from the Hanoi Medical University but restrictions apply to the availability of these data, which were used under license for the current study, and so are not publicly available. Data are however available from the authors upon reasonable request and with permission of Hanoi Medical University (contact bach.ipmph2@gmail.com).

## Abbreviations

WHO: World Health Organization
PICSS: Perinatal Infant Care Social Support
SEM: Structural equation modeling
RMSEA: Root Mean Square Error of Approximation
CFI: Comparative Fit Index
SRMR: Standardized Root Mean Square Residual
